# A Novel Strategy for Very-Large-Scale Cash-Crop Mapping in the Context of Weather-Related Risk Assessment, Combining Global Satellite Multispectral Datasets, Environmental Constraints, and In Situ Acquisition of Geospatial Data

**DOI:** 10.3390/s18020591

**Published:** 2018-02-14

**Authors:** Fabio Dell’Acqua, Gianni Cristian Iannelli, Marco A. Torres, Mario L.V. Martina

**Affiliations:** 1 Department of Electrical, Computer, Biomedical Engineering, University of Pavia, Via Adolfo Ferrata, 5, I-27100 Pavia, Italy; 2 Ticinum Aerospace S.r.l., I-27100 Pavia, Italy; gc.iannelli@ticinumaerospace.com; 3 Instituto de Ingeniería, UNAM, C.P. 04510 Ciudad de México, Mexico; mtorresp@iingen.unam.mx; 4 Scuola Universitaria Superiore IUSS Pavia, Piazza della Vittoria, 15, I-27100 Pavia, Italy; mario.martina@iusspavia.it

**Keywords:** best practice, crop mapping, crowdsourcing, drought risk assessment, exposure, flood risk assessment, geospatial data, spaceborne remote sensing, unsupervised classification, rule-based classification

## Abstract

Cash crops are agricultural crops intended to be sold for profit as opposed to subsistence crops, meant to support the producer, or to support livestock. Since cash crops are intended for future sale, they translate into large financial value when considered on a wide geographical scale, so their production directly involves financial risk. At a national level, extreme weather events including destructive rain or hail, as well as drought, can have a significant impact on the overall economic balance. It is thus important to map such crops in order to set up insurance and mitigation strategies. Using locally generated data—such as municipality-level records of crop seeding—for mapping purposes implies facing a series of issues like data availability, quality, homogeneity, etc. We thus opted for a different approach relying on global datasets. Global datasets ensure homogeneity and availability of data, although sometimes at the expense of precision and accuracy. A typical global approach makes use of spaceborne remote sensing, for which different land cover classification strategies are available in literature at different levels of cost and accuracy. We selected the optimal strategy in the perspective of a global processing chain. Thanks to a specifically developed strategy for fusing unsupervised classification results with environmental constraints and other geospatial inputs including ground-based data, we managed to obtain good classification results despite the constraints placed. The overall production process was composed using “good-enough" algorithms at each step, ensuring that the precision, accuracy, and data-hunger of each algorithm was commensurate to the precision, accuracy, and amount of data available. This paper describes the tailored strategy developed on the occasion as a cooperation among different groups with diverse backgrounds, a strategy which is believed to be profitably reusable in other, similar contexts. The paper presents the problem, the constraints and the adopted solutions; it then summarizes the main findings including that efforts and costs can be saved on the side of Earth Observation data processing when additional ground-based data are available to support the mapping task.

## 1. Introduction

Whereas subsistence crops are meant to support the producers or their livestock, cash crops are agricultural crops grown with the intended purpose of selling them for profit. Since cash crops are destined for future sale, they hold a relevant financial potential during their growth season, which is constantly in danger due to various types of threats, including weather-related threats. Among these latter threats, we should surely account extreme weather events, including:

- excess rainfall, and consequent floods and damage to crops;
- hail, destroying growing plants;
- extreme drought, leading to death of plants.

Events like those outlined above may have a significant impact on those nations whose Gross Domestic Product (GDP) is largely supported by agricultural activities. Mapping cash crops at a national, or even super-national level when regional strategies are concerned, is a necessary action to assess exposure. Thus, cash crop mapping is indeed a crucial part of any large-scale risk assessment actions intended to define a risk-mitigation—or at least an insurance—strategy to be put in place.

The work described in this paper was carried out on the Caribbean area, but designed with a global perspective in mind, in order to make it reusable anywhere else in the world. This produced the following implications:

- *avoidance of local datasets*; local datasets, such as municipality-level records of crop seeding, or surveying results from local authorities, are obviously not reusable out of their own geographical scope, but especially they may be severely inhomogeneous across different countries or regions. Global datasets were to be used, albeit at the cost of lower precision, accuracy, or resolution.
- *leveling of expected quality*; highly-refined components of the risk equation are of little use where they have to be forcibly combined with much coarser ones in the same risk computation.

Typically, when large-scale mapping of vegetation is required, a popular approach to the problem is based on spaceborne remote sensing [1], for which different land cover classification strategies are available in literature. Hyperspectral remote sensing is a good option when classification of vegetation is concerned [2].

Spaceborne remote sensing complies with the requirements set out above, as it is inherently global, and the widest range of possible classification approaches, from the coarsest to the finest, from the leanest to the most data-hungry, ensures a suitable approach can be defined for each expected quality level.

In our case, we complemented the selected classification approaches through smart fusion of unsupervised classification results with environmental constraints and other geospatial inputs including ground-based data. This paper describes the tailored strategy developed on the occasion as a cooperation among different groups with diverse backgrounds, a strategy believed to be reusable in other, similar contexts. The paper presents the problem, the constraints and the adopted solutions; it then summarizes the main findings including that efforts and costs can be saved on the side of Earth Observation data processing when additional ground-based data are available to support the mapping task. Section 2 presents the context and explains the general terms of the problem from a risk assessment perspective. The following Section 3 ushers into the specific mapping problem. Section 4 presents some possible, standard strategies for large-scale crop mapping based on multispectral spaceborne data. Section 5 illustrates the system of constraints that originate from ranges of environmental conditions suitable for the growth of each type of cash crops. Section 6 reports on how the space-based data, environmental constraints and ground-based data can be jointly exploited to map cash crops even with limited resources. Section 7 presents results and Section 8 draws some conclusions.

## 2. General Context: Global Exposure Data for Risk Assessment

At a global scale, natural disaster financing or insurance mechanisms are in big need of datasets for risk assessment, especially for developing countries where organized, systematic data collection cannot always be guaranteed due to lack of resources or structural issues (see [3,4,5]). In particular, the activation of rapid post-event financial protection needs quick and ready-to-implement response to natural hazards. The use of local data for this purpose hinders full deployment of balanced insurance coverage due to various problems: time constraints (e.g., time consumed to collect and process local data), reliability and precision issues (to what extent can a large pool of data collected by different agents be trusted?), homogeneity issues (different countries may imply different standards), and so on. In this context, leveraging global datasets could be part of the solution—global data sets, often available at a reasonable price or sometimes even for free, represent a good starting point for local assessment if combined in the correct manner with local data. Unlike for traditional catastrophe risk modelling, catastrophe models used within parametric programs by investors and/or insurers to finance disaster risks require the data be available and reliable not only for the present time, i.e., for real-time operational use, but also for historical periods. These models are used both as the basis for loss estimation in (near) real time and for risk assessment to verify solvency or to make the product quotations. Therefore, it is a fundamental requirement that the data used for both purposes are statistically homogeneous, in order to avoid biasing the analysis. Even for a conventional risk model for the Caribbean area, exposure data, i.e., geographical maps of assets exposed to threats, are a fundamental component; in our case, the exposure information consists of maps with geographical distribution of the different types of crops. To this purpose, the goal was not to generate the best exposure data in absolute terms but rather exposure data whose quality level was consistent with:

- the general model used for overall risk assessment, and
- the accuracy level of the other datasets incorporated in our production.

Given this context and the linked requirements, we came up with the following guidelines for our work:

1. it was important to balance the different components of the risk model in order to avoid mixing datasets that score too differently in terms of accuracy and precision; in this case, finer data or finer models would indeed be underused;
2. our goal was to hit the optimal trade-off between data availability and the specific needs of the vulnerability component in order to capture the main factors affecting risk.

In the following chapter, we will specify the case analyzed, and present our solution, specifically developed from the given conditions and the guidelines above.

## 3. Aims

**Figure 1:**
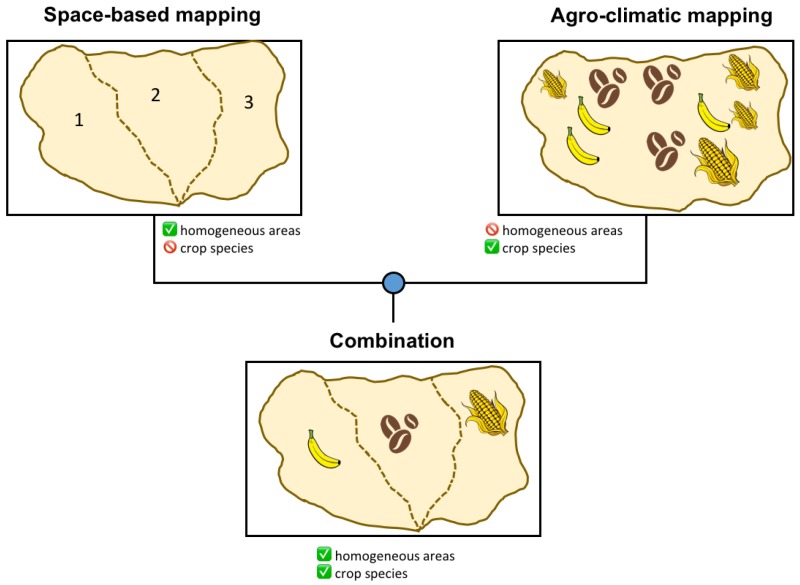
The flowchart of the proposed methodology. The box in the top left represents the output of the satellite-based mapping of homogeneous areas. Each homogeneous area is labeled with a number, but no specific crop class. The box in the top right represents the output of agro-climatic mapping, where each agro-climatic areas is associated with several possible crops, compatible with the local environmental conditions. None of these two outputs alone can directly translate into a crop map. The blue dot in the middle represents the fusion method described in Section 6, which leads to an actual, full crop map.

The main aim of the work is to map the crop areas over a large territory through the combination of two mapping techniques, each individually incomplete for the purpose. The first technique is based on spaceborne remotely sensed data that is able to cluster the territory into homogeneous areas by analyzing the satellite images over a relatively long time interval, but it is not able to identify the crop kind. The second technique is able to identify the crop kinds that potentially could grow in the territory given the agro-climatic conditions but is not able to determine which are actually grown. The combination of the space-based crop mapping and the agro-climatic mapping aims at a crop classification sufficiently accurate to provide the exposure information for weather related risk assessment. Our fusion concept is illustrated in Figure 1, where the information contribution from each branch of the confluence is visually highlighted. The second aim of the research is to make the methodology suitable to be easily applied everywhere. This implies the use of only data available at a global scale and the development of unsupervised techniques.

## 4. Space-Based Crop Mapping

### 4.1. Scientific Background

Spaceborne remote sensing is generally more efficient and effective than traditional methods, like field surveying, in describing the Earth surface. This is due to its ability to map and monitor the spatial distribution of land cover continuously and consistently at a variety of spatial and temporal scales, even though it may come at the expense of slightly worse thematic accuracy, depending on the approach used [6]. Several efforts have been made to map land cover at a global scale; some examples include: IGBP DISCover [7], UMD Land Cover [8], and Global Land Cover 2000 [9]. At a regional scale, the GMES, then Copernicus, initiative in Europe produced the Corine Land Cover (CLC) [10] map reporting land cover on the European continent, made publicly available on the Copernicus Land repository [11]. This is all but easy, given the absence—until recent times—of well-registered multi-temporal datasets, skills and processing power [12]. Focusing on vegetation and crop mapping, the problem gets more difficult [13] given the stronger similarity in spectral response among all vegetated species with respect to a situation where general categories of land cover are to be discriminated. Moreover, a high revisit frequency is needed in many areas, in order to maximize the chances of getting cloud-free data. In addition, the growing cycle of vegetation in agriculture needs fine temporal resolutions to be able to separate crops that are similar in spectral response but show temporal differences in their phenological cycle [14]. This concept has been around for several years now [15], but the difficulty of the problem (possibly also together with the scarcity of data) made research efforts to focus on detection of very few classes [16]. Even with the recent research progress favoured by the increasing richness of spaceborne data available, the problem of discriminating several different types of crops is still regarded as a difficult one [17]. MODIS [18,19,20] data and, at a finer spatial resolution, SPOT [21] data have most commonly been used for this purpose, whereas very high resolution (VHR) data is practically absent from the crop mapping context probably due to a combination of large geographical scale and high per sq. km cost of data. In our case, rather than attempting to directly classify the crop type with all the consequent issues pointed out above, we took a different approach, made possible by the availability of formalized environmental constraints on crop types (see Section 5). We indeed focussed on the simpler problem of finding homogeneous areas, defined as contiguous areas displaying homogeneous behaviour in multitemporal series of multispectal spaceborne optical data.

### 4.2. Our Approach

As mentioned above, we focused on outlining homogeneous areas rather than directly classifying crop types. As expected, single-date spectral responses are very similar to each other, so multitemporal trends had to be exploited. In this context, MODIS data was the first choice, supported by two very important strongpoints it possesses in this context:

- open availability;
- high temporal frequency (roughly every second day in the equatorial band, daily elsewhere).

Given the scarce spectral separability of the species to be mapped, temporal frequency becomes of paramount importance here as discrimination will have to rely upon phenological cycles as reflected in temporal trends of vegetation indexes. Terra and Aqua were launched in December 1999 and May 2002, respectively, as the first items in a series of multi-instrument spacecraft forming NASA’s Earth Observing System (EOS). The latter includes a science component and a data information system (EOSDIS) supporting global observations of both land and water and distributing data through its Distributed Active Archives [22]. Copernicus Sentinel-2 data, distributed through the Copernicus Open Access Hub [23], was included where deemed necessary to improve the situation in the case of poor separability of crop areas. Sentinel-2 data was used only as an integrative source because, with respect to MODIS, it has:

- lower temporal frequency; 5-days revisit time may appear very short, but in areas where cloud coverage is frequent such as the predominantly tropical Caribbean areas, the daily revisit time of Terra/Aqua can be crucial in preventing data gaps;
- higher spatial resolution, while unnecessary to the foreseen application, results in gigantic files to be stored and processed. This is only worthwhile when the additional data makes a difference in terms of separability of relevant land cover classes.

As a consequence, we selected MODIS as the main data source for our work, and Sentinel-2 as an integrative source where necessary; Table 1 summarizes the Earth Observation data types used in this work and their main features involved in our choice. Multitemporal MODIS data was thus searched on the “Level-1 and Atmosphere Archive and Distribution System” (LAADS) repository, maintained by the “Distributed Active Archive Center” (DAAC) of the “National Aeronautics and Space Administration” (NASA). Images that turned out to be too cloudy on solid land were discarded, while valid Level-1 images were retained and organized by country covered.

**Figure 2:**
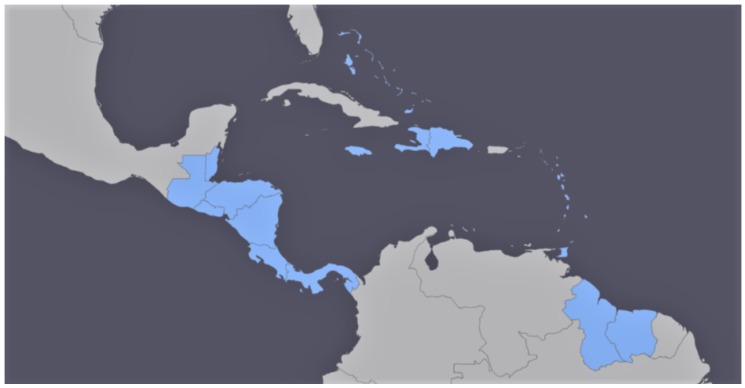
Map of the concerned area in Central and Southern America, North up. Countries included in our analysis are represented in light blue, whereas other solid land is represented in gray and oceans in dark, bluish gray.

**Figure 3:**
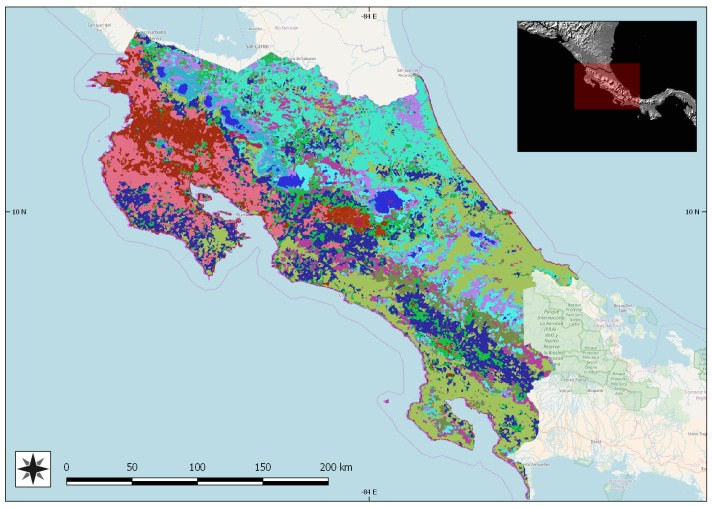
Sample results on Costa Rica. Each distinct color depicts one region reputed to be homogeneous according to the clustering method described.

The geographic scope of the contracted study, highlighted in blue in Figure 2, was ample both in latitude and longitude. Thus, one of the first issues was to ensure geographical consistency of the multitemporal datasets and seamless merging of adjacent tiles on land; the ocean surface is not relevant in a vegetation-related study. This entailed welding together several tiles. An automated procedure based on SIFT [24] involving identification and matching [25] of feature points [26] was developed [27] considering the size and characteristics of MODIS images, with special attention to geometric consistence; uniformity of control points was not a priority, considering that in many cases most of the imaged area was ocean surface. Such methodology was then applied to the downloaded, dense time series. Next, cloudy areas were removed using an algorithm specifically developed for large-scale multispecral datasets [28,29]. Once cloud masks had been built and applied, several vegetation-related indexes were extracted from multispectral data, such as NDVI, tasseled cap, LAI, and others, in order to capture as many nuances as possible in the spectral behaviour of each vegetated species. No registration was needed at a later stage between images and masks/DEMS as the result of fusing images appeared to match, with sufficient accuracy, the other geospatial data used at later stages. The following step consisted of filling in the gaps in the time series left as a consequence of cloud mapping. This was accomplished by estimating missing values with an interpolation approach based on observed series in past sequences; the current series with gaps was compared with past sequences in the geographical neighbourhood. The most similar local series from the past was detected and the value corresponding to the gap was used in place of the missing value. Once the series were complete, unsupervised clustering was applied to them, in the form of *k*-means classification applied to the time series arranged in vectorial form. This resulted in a partition of the land areas into homogeneous regions. An urban mask obtained by classification of the multispectral dataset [30] and a ready-to-use water mask also derived from satellite data [31] allowed us to remove most of the non-vegetated areas. This was the unsupervised part of the procedure, followed by a step including human supervision. Boundaries between homogeneous areas obtained through the previously mentioned unsupervised clustering were automatically extracted and visually compared with true-color satellite images in order to check their consistency. Where discontinuities apparent in true-colour satellite images did not match the above mentioned boundaries, we assumed insufficient separability of classes. These latter cases were tackled by downloading additional multispectral data at higher resolution (LANDSAT or, where available, Sentinel-2 data) to increase spectral (and temporal) separability, and clustering was repeated on the enriched dataset. This turned out to be decisive in some cases, but not many overall as only a dozen dubious boundaries had to be treated with additional data download. A further double-check step was added, consisting of an extensive search for georeferenced ground pictures published on the web. Pictures of crops were selected among those whose time stamps reported the same year/growth season as the analyzed satellite data. Where a region generated by clustering contained pictures with different crop species, such region was treated as a case of insufficient separability, and tackled using a similar approach to the one described above for inconsistent region boundaries. Only few cases of inconsistence were however observed, and the vast majority (around 98%) of checks resulted in confirmation of the first results. A final, sample check was carried out on the result to confirm that no contrary evidence emerged on homogeneity of the extracted regions. Figure 3 shows a sample result on Costa Rica.

## 5. Agro-Climatic Mapping

As it was mentioned in Section 4.1, the problem of discriminating several different types of crops directly from spaceborne data requires the analysis of spectral responses and phenological cycles of multi-temporal datasets with very high time-frequency and resolution. This can be solved in some way when mapping small areas, but it can be very complex, expensive and demanding if we combine large geographical scale, high per sq. km cost of data, and constraints on time and resources.

**Figure 4:**
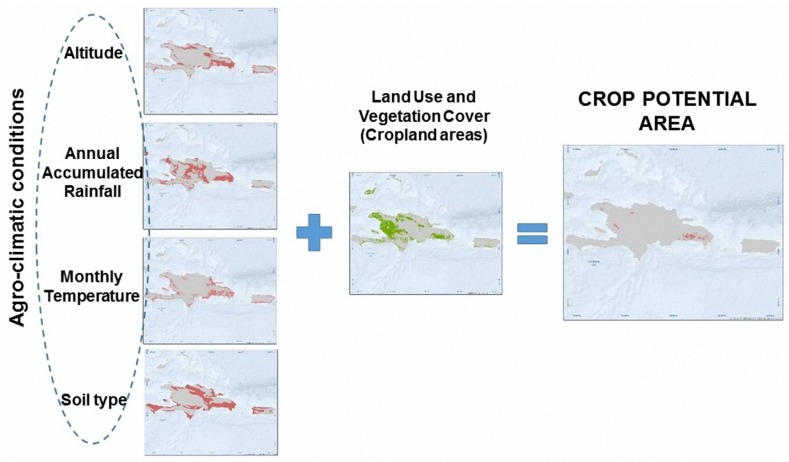
Schematic core process of agro-climatic mapping.

The methodology described in the Section 4.2 is able to classify the territory into spectrally homogeneous areas allowing to separate areas with very different spectral responses (e.g., urban areas and forest areas) and group the ones with very similar response (e.g., the croplands with similar spectral response). However, the spaceborne mapping technique, as previously illustrated, is not able to attribute to each spectral class a specific crop type (coffee, banana, rice, etc.). Therefore, a second independent technique of classification has been implemented. The agro-climatic mapping identifies for each unit of the territory the crops that potentially could grow according to their aptitude and potential agricultural production with respect to the actual environmental conditions [32]. The approach used in this chapter focuses on the evaluation of an area of interest and according to the agro-climatic conditions required for the selected crop, in order to estimate potential areas for its cultivation. Finally, these potential areas are confined into the areas classified as croplands through public products of land use and vegetation derived from spatial sensors. A schematic illustration of conceptual agro-climatic mapping is shown in Figure 4.

The final aim of the procedure described in this chapter is to obtain maps, for each country, where the areas classified as croplands are assigned potentials of production of certain types of crops. To carry out this mapping technique, two processes are basically performed:

1. Definition of the agro-climatic conditions required for each crop to achieve its potential production through the regression analysis of reference data from public sources. The parameters that define the agro-climatic conditions are: (1) annual precipitation, (2) monthly temperature (minimum and maximum), (3) elevation over sea level, and (4) edaphology.
2. Estimation of crop potential areas that are those cropland areas where all the ranges of agro-climatic conditions are fulfilled. In this step, the agro-climatic parameters defined previously and the cropland classification are contained in different layers that are spatially crossed to unify all in a single element [33].

### 5.1. Input Data

To perform the agro-climatic mapping, we needed data about elevation above sea level, monthly average temperature (°C), annual average precipitation (mm) and units of dominant soils (edaphology) for the entire region. For many countries, no information on the selected crops could be found; in these cases, the climatological conditions were estimated based on the data of neighboring countries or regions presenting similar climatic characteristics.

The temperature and precipitation data were downloaded from the WorldClim’s database, which offers a wide range of monthly precipitation data, as well as monthly minimum and maximum average temperature data with a resolution of 30 s or 1 km approximately [34,35]. The elevation data downloaded was recorded by the sensor Shuttle Radar Topography Mission (SRTM), which was on board the Endeavour shuttle [36]. The soil data was obtained from the FAO’s database, which offers the Digital Soil Map of the World (DSMW) in shape format at a scale of 1:5,000,000 with a detailed report by global regions about types and soil mapping methods. Table 2 provides a brief summary of the characteristics of the input data used.

### 5.2. Definition of Agro-Climatic Conditions

Conditions for the same crop can even variate from one country to another. With the aim of knowing the different geo-climatic conditions of each crop in each region, an exhaustive search of reference information was performed to locate main areas of the different cash crops. Generally, these cartographic data are not very detailed, neither when obtained from remote sensing, nor from site inspections. Basically, they consist of schematic maps with general polygons without a scale or an adequate resolution obtained from national sources, for example, the Protected Areas Conservation Trust in Belize; the Ministry of Agriculture, Natural Resources and Rural Development in Haiti; the System of Information of Forest Resources of Costa Rica and the Guyana Lands and Surveys Commission, among others; and from global sources such as the United States Agency for International Development (USAID); the United States Geological Survey (USGS); the Famine Early Warning System Network (FEW Net); the Food and Agriculture Organization (FAO); the World Bank and the Interamerican Development Bank (BID).

**Figure 5:**
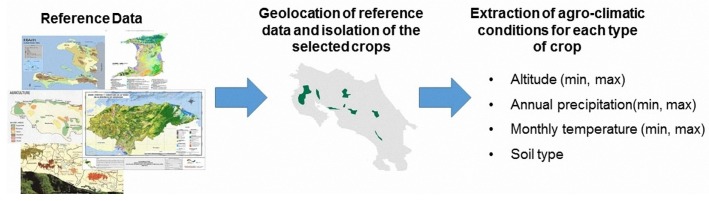
Definition of agro-climatic conditions through the regression analysis of reference data used in this study .

As is shown in Figure 5, the reference data is geolocated and classified to isolate the areas of interest in raster or vector format. The obtained layer is useful as a mask to outline and extract the agro-climatic conditions according to each selected crop and from a spatial combination between these conditions obtaining the potential areas in which a crop is cultivated. When the reference data is available, we use the range of conditions obtained in that country and we extrapolate the conditions for similar climatological countries without reference data of the crops.

**Figure 6:**
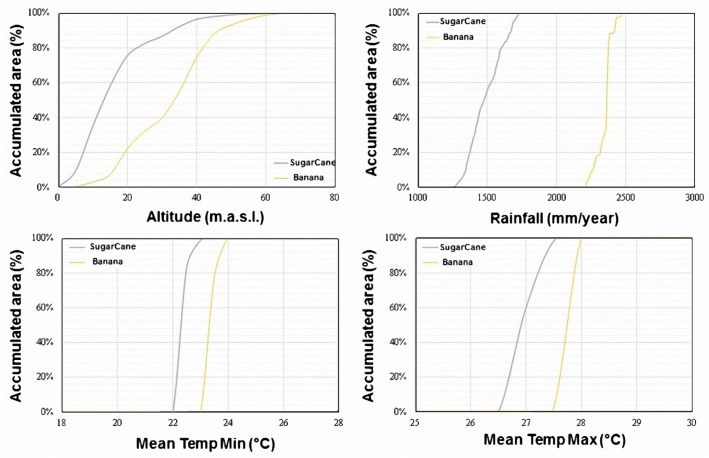
Sample of curves of accumulated area in percentage estimated for two types of crops in Belize.

To define the geo-climatic conditions through the masks of the reference areas, the climatic data along with elevation data was spatially crossed with the reference areas in order to establish the maximum and minimum value inside those areas. These ranges of the agro-climatic variables for each crop and country was obtained by means of curves of accumulated area (CAA). In the CAA, the *x*-axis represents the values of the agro-climatic variable and the *y*-axis represents the accumulated area up to a certain value of the agro-climatic variable, therefore, a 100% in the *y*-axis corresponds to the total area of the reference map used. The CAA were computed as follows: at first (1) to filter the agro-climatic layers through the mask of the reference maps, then (2) to obtain the histogram of areas through counting the areas/pixels associated to agro-climatic values, and finally (3) to accumulate the areas of the histogram, in terms of percentage, according to the values of the agro-climatic variable. In Figure 6, we show as a sample the CAA obtained for Belize for the selected cash crops. In this study, the geo-climatic intervals were selected considering at least 95% of the accumulated area of the reference masks. Table 3 shows, as a sample, the agro-climatic conditions obtained in Costa Rica for the five types of crops considered: banana, coffee, maize, rice and sugar cane.

### 5.3. Estimation of Crop Potential Areas

As it is shown in Figure 4, once the range of values of the agro-climatic conditions were defined from the accumulated area curves, we verified, in the different agro-climatic layers (altitude, monthly temperature, annual precipitation, and dominant soil type), that the value of each pixel be within the ranges; if this was the case, we assigned a value of 1; in a contrary case, we assigned a value of 0. Finally, we computed the product among the *binary layers*, obtaining a single layer that represents with a value of 1 the pixels/areas where the agro-climatic conditions are fulfilled.

In order to limit the analysis to agricultural/vegetation areas, we sought cropland area maps that could be used as a mask, discarding classes without vegetation: urban, water bodies or any other class having no relation to agriculture. The selected product was *MODIS MCD12Q1 (Land Cover Type Yearly L3 Global 500 m),* which supplies global maps of land cover at annual time steps and 500 m spatial resolution for 2001 to 2013 [37] (Table 4).

The *MCD12Q1* classification incorporates five different land cover class schemes, derived through a supervised decision-tree classification method [38]. In addition, an assessment of the relative classification quality (scaled from 0–100) is provided at each pixel, along with quality assurance information and an embedded land/water mask. The five classification schemes are as follows:

1. The International Geosphere Biosphere Programme (IGBP) scheme, in which seventeen land covers were identified, eleven of them being vegetation, three terrain classes and three more vegetation-free classes. Their stated objective was to provide a global land cover dataset that was more up-to-date, of known accuracy and with higher spatial resolution and greater internal consistency than any other existing dataset. The scheme is based on definitions of three canopy components: above-ground biomass, leaf longevity, and leaf type process. The land-cover categories identified by the IGBP are related to the needs of gas exchange studies; vegetation attributes for modeling Net Primary Production (NPP); burn emissions and gas exchange; wetlands cover and wetland water regimes; changes in vegetation/land-cover over time; biological attributes; physical attributes, and landscape characteristics [38,39,40,41].
2. The University of Maryland land cover classification (UMD) dataset, with fourteen classes, two of which have no vegetation. The approach taken involved a hierarchy of pairwise class trees where a logic based on vegetation form was applied until all classes were depicted. Minimum annual red reflectance, peak annual Normalized Difference Vegetation Index (NDVI), and minimum channel three brightness temperature were among the most used multitemporal metrics. Depictions of forests and woodlands, and areas of mechanized agriculture are in general agreement with other sources of information, while classes such as low biomass agriculture and high-latitude broadleaf forest are not [8].
3. The LAI/FPAR scheme, with nine vegetation classes and two vegetation- free ones. This scheme uses a method for the estimation of global leaf area index (LAI) and fraction of photosynthetically active radiation absorbed by the vegetation (FPAR) from atmospherically corrected Normalized Difference Vegetation Index (NDVI) observations. LAI is defined as the one-sided green leaf area per unit ground area in broadleaf canopies and as one half of the total needle surface area per unit ground area in coniferous canopies. FPAR is defined as the fraction of incident photo-synthetically active radiation (400–700nm) absorbed by the green elements of a vegetation canopy. The method requires stratification of global vegetation into cover types that are compatible with the radiative transfer model [42].
4. The Net Primary Production scheme (NPP) with nine vegetation classes and two vegetation-free ones. NPP defines the rate at which all plants in an ecosystem produce net useful chemical energy. In other words, NPP equals the difference between the rate at which plants in an ecosystem produce useful chemical energy (or GPP, Gross Primary Production), and the rate at which they expend some of that energy for respiration. The Primary Production products are designed to provide an accurate regular measure of the growth of the terrestrial vegetation. Version-55 Terra/MODIS NPP products are validated to Stage-3; this means that its accuracy was assessed and uncertainties in the product were well-established via independent measurements made in a systematic and statistically robust way that represents global conditions. These data are deemed ready for use in science applications [43].
5. The Functional Type Plant scheme (FTP) with nine vegetation classes, two vegetation-free and one ice-water class. While most land models developed for use with climate models represent vegetation as discrete biomes, this is, at least for mixed life-form biomes, inconsistent with the leaf-level and whole-plant physiological parameterizations needed to couple these bio-geophysical models with bio-geochemical and ecosystem dynamics models. In the calculation of this scheme, the authors present simulations with the National Center for Atmospheric Research land surface model (NCAR LSM) that examined the effect of representing vegetation as patches of plant functional types (PFTs) that coexist within a model grid cell. This approach is consistent with ecological theory and models and allows for unified treatment of vegetation in climate and ecosystem models [44].

The different classes included in the classification schemes are shown in Table 5. As can be seen, four of the five classification schemes include categories referring to agriculture, except the *NPP* scheme that does not include any class related to croplands; therefore, it was not considered in the process. The classes corresponding to each crop are obtained and combined according to its leaf morphology. The obtained combinations for each one of the selected crops are listed in Table 6. To avoid inconsistencies among the classification schemes, we assume that an area was considered as a cropland only if at least three out of the four considered schemes agreed on it being a cropland area. Similar to the agro-climatic conditions, we obtained a final binary layer that represents the existence of detected croplands. This layer was also spatially crossed with the layer of agro-climatic conditions, thus obtaining the final map of potential crop areas.

## 6. Information Fusion Strategy

The spectrally homogeneous areas extracted from satellite imagery (method described in Section 4) and the crop potential areas (method described in Section 5) are individually insufficient to identify the location of the selected crops for purposes of risk assessment. Fusion of both approaches leads to establishing a relationship between the agro-climatic requirements of potential crops with their spectral responses in situ, thus opening the doors for mapping at a very large scale.

The main aim of the fusion strategy consists of assigning to each identified spectrally homogeneous region the most likely one among all the locally possible crops. This fusion process was carried out using all the data information in raster format, so the analysis of the homogeneous areas were performed through the analysis of the pixels inside the agro-potential areas of each entire country. In order to identify more clearly the membership of an area/pixel to one type of crop or another, the identification process was firstly performed in the areas without agro-potential uncertainty (just one type of crop with agro-potential in that area), and then the identified homogeneous classes were applied to the areas/pixels with agro-potential uncertainty (two or more crops sharing the same potential area).

The first criteria to classify a homogeneous class was the potential crop type with the higher percentage of suitable sub-area for the areas without uncertainties. After obtaining the homogeneous classes related to each type of crop, we merged the areas/pixels with uncertainties, and then we summed the areas covered by the different selected homogeneous classes to estimate the amount of covered area of each crop, making sure at the same time that they were close to the reported statistics of harvested area (in this study, the data from the Food and Agriculture Organization), and that the identified areas covered the checkpoints.

In cases where the obtained total area was far below the reported statistics, we returned to the first step and included the following homogeneous class with higher area percentage to the analyzed crop, provided it was not selected for another crop, and repeat the process until we were close to the target. In this case, the group of homogeneous classes selected were labeled as the analyzed crop. In the contrary case, when the total labeled area was far above the statistic, we discarded the selected homogeneous class and chose another class with smaller coverage area and repeated the process.

Crop areas are mutually exclusive, so if an homogeneous class or a group of homogeneous classes were labeled as a certain type of crop, they can not be labeled as another type. Once the assignation was completed and the conditions had been verified, the next step was to extract from the homogeneous areas the assigned classes and create the final map of crop areas.

A test was carried out on the region of San Miguel, El Salvador, which was first analyzed using high-resolution satellite imagery processing through fusing two different sources of multispectral spaceborne remote sensing images (i.e., Landsat-8 and Sentinel-2) and crowdsourcing data (e.g., in situ georeferenced photos).

**Figure 7:**
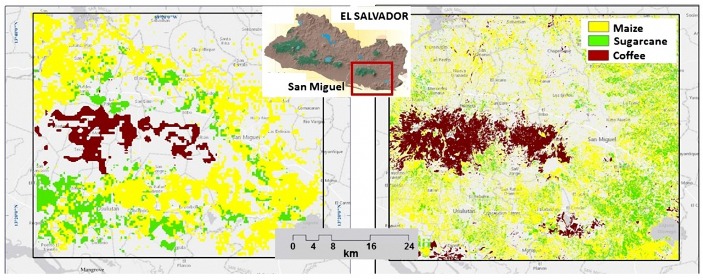
Areas estimated through the proposed approach (**left**) and the high-resolution analysis (**right**) on the region of San Miguel, El Salvador.

In Figure 7, it is shown the accurate thematic map obtained through the complex analysis of high-resolution imagery in comparison with the output of the proposed approach. As it can be noticed, the results of the proposed approach are very similar to those obtained from the costly high-resolution analysis.

## 7. Results and Validation

### 7.1. Results of Estimated Areas

**Figure 8:**
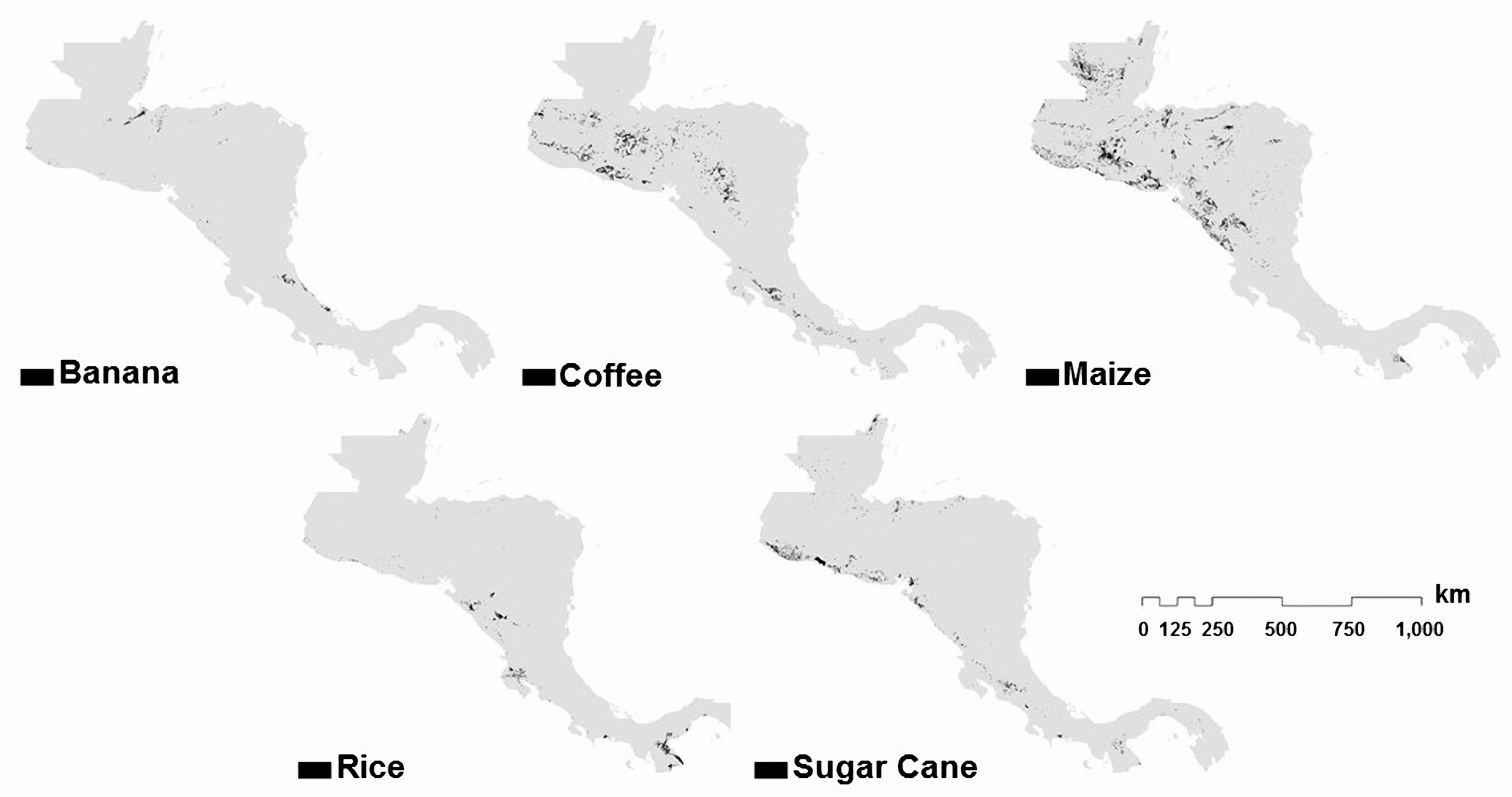
Estimated areas of the selected crops in the region of Central America.

**Figure 9:**
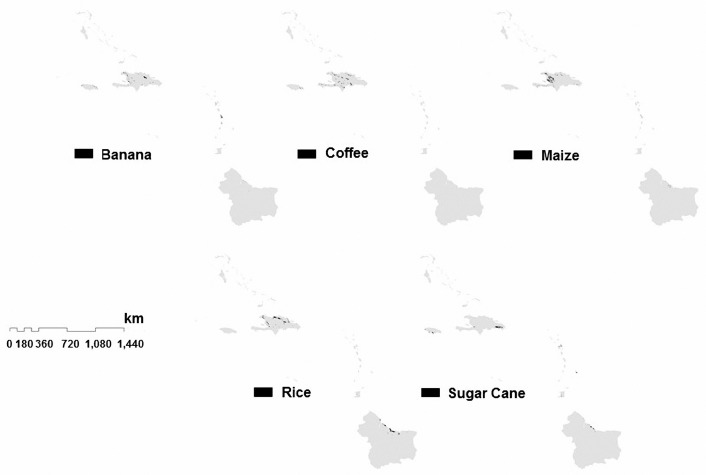
Estimated areas of the selected crops in the region of the Caribbean.

The approach described in Section 6 is used to estimate the areas of five different cash crops (banana, coffee, maize, rice, and sugar cane), selected due to their economical importance in the region. The results of the estimated areas are shown in Figure 8 and Figure 9.

### 7.2. Verification with Checkpoints

**Figure 10:**
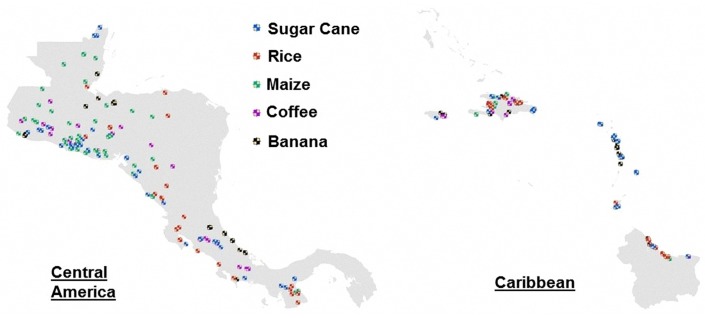
Checkpoints with crowdsourced images in the regions of Central America (**left**) and the Caribbean (**right**) used to validate the obtained areas.

**Figure 11:**
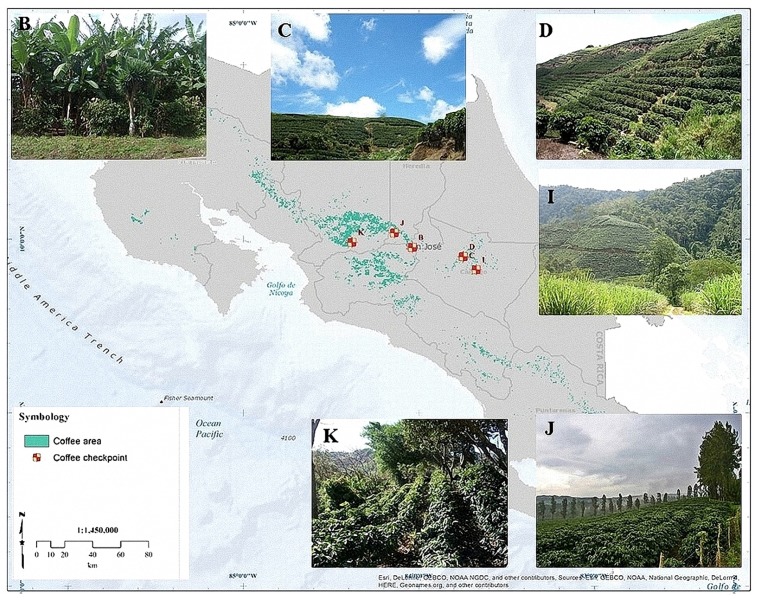
Sample of estimated areas of coffee and verification through checkpoints from crowdsourcing’s images in Costa Rica.

The final areas obtained for each crop are spatially compared with checkpoints and the reference areas from Section 5.1. The search of the checkpoints is done through crowdsourcing from the site *Panoramio*, owned by Google (Mountain View, CA, USA), which possesses a database of geo-located tag photos uploaded by users around the globe. Panoramio only allows outdoors photos, which is advantageous to us because it implies removing a lot of data clutter. Every time a user uploads a picture, it is revised for compliance with this criterion, which ensures we only find relevant pictures when searching in agricultural areas. The site could be accessed as a layer in *Google Earth* and *Google Maps*. The total number of checkpoints used in the region can be seen in Figure 10, which were distributed in the following way: 65 for banana, 33 for coffee, 56 for maize, 58 for rice and 82 for sugar cane. As a sample of the verification of the results through the checkpoints, in Figure 11, the estimated areas for coffee in Costa Rica are shown, including the location and images of the checkpoints used to validate the results.

### 7.3. Comparison of Estimated Areas vs. FAO’s Statistics

**Figure 12:**
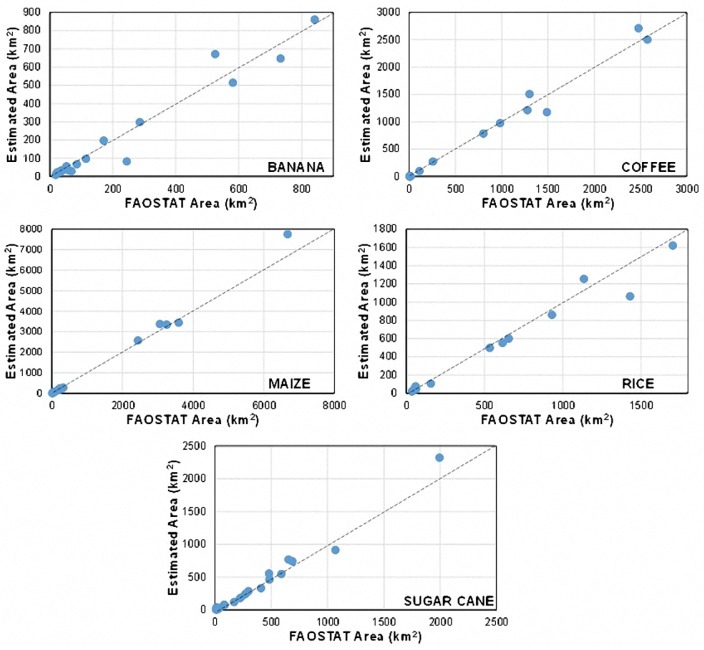
Comparison between estimated area vs. FAO’s statistics in the considered countries.

In order to have both spatial and numerical certitude, the final areas were compared with the statistics of the harvested area of the countries of the region to verify that the error be under acceptable levels. As it can be seen in Figure 12, where each point represents the estimated area vs. the statistics reported from FAO (Food and Agriculture Organization) of each country considered, the estimated areas per country are very close to the data reported in the FAO’s statistics. In the same way, in Table 7, the error per crop area in percentage using the estimated areas and the FAO’s statistics in the entire region is shown, and, in all the selected crops, the difference is smaller than 10%. This step is crucial since the level of precision required in a study of agricultural drought risk assessment.

## 8. Conclusions

In this work, we present a methodology to map cash crops on a large geographical scale, for purposes of weather-related risk assessment. Cash crops are agricultural crops grown to be sold for profit, so losses on cash crops due to weather-related events such as flood, drought, and hail, can produce a large financial impact. Such impact may be especially dangerous for countries that strongly depend on their production like the Caribbean islands and Central American countries, so risk assessment and consequent insurance is necessary to keep those countries somehow safer from the financial effects of extreme weather events.

A founding part of risk assessment is exposure mapping, i.e., mapping the spatial distribution of resources that are at stake. Mapping agricultural resources on a large area such the one addressed in this study may be very costly and may weigh excessively on the overall balance of cash crop businesses.

Confronted with this problem, we developed a novel technique for large-scale crop mapping, largely relying on open data and algorithms offering low computational burdens. Specifically, two mapping techniques were merged, unsupervised classification on time-series of multispectral data, and climate-based zoning. Each of these techniques individually would be insufficient for cash crop mapping, but a suitable combination leads to our goal while curbing the cost of data procurement and processing. The space-based mapping technique clusters’ homogeneous crop areas, i.e., areas showing an evolution in time of vegetative parameters that is “sufficiently homogeneous” within each region and “sufficiently different” across regions to conclude that areas of homogeneous crop species were identified. The agro-climatic technique can instead identify crops potentially grown at each different environmental condition. Each technique individually provides results with a certain degree of ambiguity, but merging information from both results in sensible crop maps, through cross-resolution of conveyed ambiguities.

We tested the developed methodology over the Caribbean and Central America countries. The resulting cash crop maps have been validated both visually and by comparison with FAO’s data. On 294 different checkpoints and reference areas, the comparison of the mapped crops with geo-referenced pictures available via Google Panoramio showed very good agreement. In order to provide an additional assessment of the quality of results, FAO’s data of the harvested area by countries and crops were compared with mapping results, showing mismatch figures below 10%. This mismatch level is considered sufficient for risk assessment purposes because weather threats to crop yield (droughts, excess of rain, tropical cyclones, etc.) tend to affect medium- to large-sized areas, thus averaging out local classification errors.

With respect to other possible approaches to large-scale crop mapping, the main advantages of the proposed method are its lower cost (i.e., less manpower required while still maintaining high standards of accuracy) lower computational burden and its flexibility (geo-spatial evidence from other sources than satellite data, with special reference to crowdsourced pictures which may help to correctly label each homogeneous area).

In the context of risk assessment, the importance of exposure data is fundamental. However, the accuracy and resolution of these data should be coherent with the purposes and the requirements of the risk model applied. Often, the approximations involved in the vulnerability or hazard component of the model would make overcast the accuracy and the resolution of exposure crop data. The developed methodology allows for a direct application on other territories since it only makes use of globally accessible and available data and of unsupervised automatic techniques.

## Figures and Tables

**Table 1:** Spaceborne Earth Observation (EO) data types used in this work, and their respective roles.

Spaceborne EO System	Ground Resolution (m)	Swath Width (km)	Revisit Time (days)	Usage in Project
MODIS	250–1000	2330	6	baseline
Sentinel-2	10–60	290	2–3	occasional

**Table 2:** Description of data used to define agro-climatic conditions.

Database	Variables	Ground Resolution	Scale/Applicability	Period
WORLD CLIM	monthly minimum, mean and maximum temperature, precipitation, solar radiation, wind speed and water vapour pressure	30 s (∼1 km^2^) to 10 min (∼340 km^2^)	Global, Regional, National, Sub-national, Province/District, Watershed/Basin, Landscape	Average monthly for 1970–2000
SRTM	elevation data	1 s (∼30 m)	Global, Regional, National, Sub-national, Province/District, Watershed/Basin, Landscape	2000
DSMW	soil data	5 min (∼170 km^2^)	Global, Regional, National	Since 1961

**Table 3:** Ranges of values of agro-climatic conditions estimated for the selected crops in Costa Rica.

*Crop*	*Altitude* *(m.a.s.l.)*		*Temperature* *(°C)*		*Precipitation* *(mm/year)*		*Dominant Soil*
	*Min*	*Max*	*Min*	*Max*	*Min*	*Max*	
*Banana*	*2*	*130*	*24.0*	*28.0*	*2800*	*4900*	*Vitric Andosols,* *Eutric Nitosols,* *Mollic Andosols*
*Coffee*	*550*	*1950*	*15.1*	*25.0*	*1800*	*4100*	*Vitric Andosols,* *Mollic Andosols,* *Eutric Nitosols*
*Maize*	*20*	*985*	*22.5*	*28.0*	*1790*	*3190*	*Eutric Nitosols,* *Dystric Cambisols,* *Eutric Gleysols*
*Rice*	*6*	*800*	*25.3*	*28.6*	*1550*	*4700*	*Eutric Nitosols,* *Pellic Vertisols,* *Dystric Ambisols*
*Sugar Cane*	*550*	*1800*	*17.5*	*29*	*1550*	*3500*	*Eutric Nitosols,* *Vitric Andosols,* *Eutric Gleysols*

**Table 4:** Dataset characteristics of MCD12Q1.

**Temporal coverage (V051)**	2001–2013
**Earth-gridded tile area**	∼1200 × 1200 km (∼10${}^{{}^{\circ}}$ × 10${}^{{}^{\circ}}$ at the equator)
**Image dimensions**	2400 × 2400 rows/columns
**File size**	∼88 MB
**Resolution**	500 meters
**Projection**	Sinusoidal
**Data type**	8-bit unsigned integer
**Data format**	HDF-EOS
**Science Data Set (SDS) layers**	16

**Table 5:** Land cover descriptions of MCD12Q1.

	*IGBP*	*UMD*	*LAI/FPAR*	*NPP*	*PFT*
*0*	*Water*	*Water*	*Water*	*Water*	*Water*
*1*	*Evergreen* *Needleleaf* *forest*	*Evergreen* *Needleleaf* *forest*	*Grasses/Cereal* *crops*	*Evergreen* *Needleleaf* *vegetation*	*Evergreen* *Needleleaf* *trees*
*2*	*Evergreen* *Broadleaf* *forest*	*Evergreen* *Broadleaf* *forest*	*Shrubs*	*Evergreen* *Broadleaf* *vegetation*	*Evergreen* *Broadleaf trees*
*3*	*Deciduous* *Needleleaf* *forest*	*Deciduous* *Needleleaf* *forest*	*Broadleaf crops*	*Deciduous* *Needleleaf* *vegetation*	*Deciduous* *Needleleaf* *trees*
*4*	*Deciduous* *Broadleaf* *forest*	*Deciduous* *Broadleaf* *forest*	*Savanna*	*Deciduous* *Broadleaf* *vegetation*	*Deciduous* *Broadleaf* *trees*
*5*	*Mixed* *forest*	*Mixed* *forest*	*Evergreen* *Broadleaf* *forest*	*Annual* *Broadleaf* *vegetation*	*Shrub*
*6*	*Closed* *shrublands*	*Closed* *shrublands*	*Deciduous* *Broadleaf forest*	*Annual grass* *vegetation*	*Grass*
*7*	*Open* *shrublands*	*Open* *shrublands*	*Evergreen* *Needleleaf forest*	*Non-vegetated land*	*Cereal crops*
*8*	*Woody* *savannas*	*Woody* *savannas*	*Deciduous* *Needleleaf* *forest*	*Urban*	*Broad-leaf* *crops*
*9*	*Savannas*	*Savannas*	*Non-vegetated*		*Urban and* *built-up*
*10*	*Grasslands*	*Grasslands*	*Urban*		*Snow and ice*
*11*	*Permanent* *wetlands*				*Barren or* *sparse vegetation*
*12*	*Croplands*	*Croplands*			
*13*	*Urban and* *built-up*	*Urban and* *built-up*			
*14*	*Cropland/Natural* *vegetation mosaic*				
*15*	*Snow and ice*				
*16*	*Barren or* *sparsely vegetated*	*Barren or* *sparsely vegetated*			

**Table 6:** Land cover combinations used for each crop.

*Crop*	*IGBP*	*UMD*	*LAI/FPAR*	*FTP*
*Maize,* *Rice*	*Croplands,* *Cropland/Natural* *vegetation mosaic*	*Croplands*	*Cereal crops,* *Broadleaf crops*	*Cereal crops,* *Broadleaf crops*
*Banana,* *Sugar Cane*	*Croplands*	*Croplands*	*Broadleaf crops*	*Broadleaf crops*
*Coffee*	*Evergreen* *Broadleaf forest,* *Cropland/Natural* *vegetation mosaic,* *Woody savannas*	*Evergreen* *Broadleaf forest,* *Woody savannas*	*Evergreen* *Broadleaf forest,* *Savanna*	*Evergreen* *Broadleaf trees,* *Deciduous Broadleaf trees*

**Table 7:** Error between estimated area vs. FAO’s statistic in the region of study.

*Crop*	*Estimated* *(km^2^)*	*FAOSTAT* *(km^2^)*	*Error* *(%)*
*Banana*	*3974.38*	*3736.30*	*6.37*
*Coffee*	*11,300.73*	*11,295.11*	*0.05*
*Maize*	*19,888.6*	*21,347.7*	*6.84*
*Rice*	*7354.98*	*6744.94*	*9.04*
*Sugar Cane*	*6656.3*	*6914.6*	*3.74*

## Abbreviations

The following abbreviations are used in this manuscript:

DAAC	Distributed Active Archive Center
EOS	Corine Land Cover
EOS	Earth Observing System
EOSDIS	Earth Observing System Data and Information System
FAO	Food and Agriculture Organization
FTP	Functional Type Plant
GIS	Geographic Information System
GMES	Global Monitoring for Environment and Security
IGBP	International Geosphere-Biosphere Programme
LAADS	Level-1 and Atmosphere Archive and Distribution System
LAI	Leaf Area Index
MODIS	Moderate Resolution Imaging Spectroradiometer
NASA	National Aeronautics and Space Administration
NDVI	Normalized Differential Vegetation Index
SIFT	Scale-Invariant Feature Transform
SPOT	Système Pour l’Observation de la Terre (System for Earth Observation, in French)
SRTM	Shuttle Radar Topography Mission
UMD	University of Maryland Department of Geography
